# Host-Parasite Interactions and Purifying Selection in a Microsporidian Parasite of Honey Bees

**DOI:** 10.1371/journal.pone.0147549

**Published:** 2016-02-03

**Authors:** Qiang Huang, Yan Ping Chen, Rui Wu Wang, Shang Cheng, Jay D. Evans

**Affiliations:** 1 State Key Laboratory of Genetic Resources and Evolution, Kunming Institute of Zoology, Chinese Academy of Science, Kunming, 650223, China; 2 USDA-ARS Bee Research Laboratory, BARC-East Building 306, Beltsville, Maryland, 20705, United States of America; 3 Center for Ecological and Environmental Science, Northwestern Polytechnical University, Xi’an, 710072, China; 4 Chongqing Academy of Animal Science, Chongqing, 402460, China; San Diego, UNITED STATES

## Abstract

To clarify the mechanisms of *Nosema ceranae* parasitism, we deep-sequenced both honey bee host and parasite mRNAs throughout a complete 6-day infection cycle. By time-series analysis, 1122 parasite genes were significantly differently expressed during the reproduction cycle, clustering into 4 expression patterns. We found reactive mitochondrial oxygen species modulator 1 of the host to be significantly down regulated during the entire infection period. Our data support the hypothesis that apoptosis of honey bee cells was suppressed during infection. We further analyzed genome-wide genetic diversity of this parasite by comparing samples collected from the same site in 2007 and 2013. The number of SNP positions per gene and the proportion of non-synonymous substitutions per gene were significantly reduced over this time period, suggesting purifying selection on the parasite genome and supporting the hypothesis that a subset of *N*. *ceranae* strains might be dominating infection.

## Introduction

Microsporidia are unicellular spore-forming intracellular parasites within the fungal lineage, parasitizing a broad range of vertebrate and invertebrate hosts [1]. The outer proteinaceous and inner chitin structured cell wall layers protect the spores against environmental stress for long term survival [2]. Spores germinate in response to proper pH and ionic condition in the midgut and inject the infective sporoplasm into the host cells with extruded polar filament. Cell proliferation starts from meronts, leading to daughter cells (sporonts) and finally the formation of mature spores [1–5]. Infected cells are filled with spores later during infection and the cell may burst to release the spores. The spores could infect neighboring epithelia cells or be expelled through feces to infect new individuals. Microsporidia parasites generally have compact genomes [1,6–8]. Classic mitochondria are absent save for tiny mitochondrially derived organelles called mitosomes, suggesting that the parasite fuels its own fast proliferation with energy from the host [9]. At the same time, the parasite needs to complete at least one replication cycle, before killing the infected host [10,11]

*Nosema ceranae* is an emerging microsporidian parasite of the European honey bee, *Apis mellifera*. After becoming infected by *N*. *ceranae*, honey bees consume more sugar solution, which suggests energetic parasitism [12]. Infection is also reported to reduce life span and suppress the immune response [13,14]. A survey of selected set of genes in *N*. *ceranae* suggests that this parasite is highly diverse [15] and the parasite is hypothesized to suppress apoptosis of infected cells for its own reproductive advantage [10]. While prior work has measured consequences of *Nosema* infection on the host [16], the mechanisms used by the parasite to manipulate host cells to establish infections are unclear. In order to study selection on *N*. *ceranae* protein-coding genes and to identify putative “virulence genes”, we infected the honey bees with *N*. *ceranae* spores and deep sequenced transcripts of both host and parasite at 24 hour intervals for the complete infective cycle (6 days post infection). Through a simultaneous analysis of both host and parasite responses during distinct infection stages and genetic diversity analysis at the genome level, we are able to provide new insights into mechanisms of microsporidia pathogenesis.

## Material and Methods

### Ethics statement

The apiaries for bee sample collection are the property of the USDA-ARS Bee Research Laboratory, Beltsville, Maryland, USA. No specific permits were required for the described studies. Studies involved the European honey bee (*Apis mellifera*), which is neither an endangered nor protected species.

### Infection and sampling

*N*. *ceranae* spores were isolated from worker honey bees in heavily infected colonies in 2013. The midguts of infected workers were homogenized in distilled water, filtered through Whatman filtering paper and centrifuged at 3220 g for 10 min. The pellet was purified using a Percoll gradient procedure [7]. Spores were counted using a Fuchs-Rosenthal haemocytometer and the *N*. *ceranae* species status was verified by a standard PCR protocol [17]. One hundred fifty freshly emerged workers were individually fed with 2 μl 50% sucrose solution containing 10^5^ *N*. *ceranae* spores. An additional 150 freshly emerged workers were individually fed with 2 μl 50% sucrose solution without spores, as a control. Each set of 50 workers were housed in a sterile plastic cup at 34 ± 1°C, 60% relative humidity [18]. In total, three cups of infected workers and three cups of control workers were constructed respectively. Five workers were sampled from each cup at 24 hour intervals for six days post-infection. After being anesthetized with CO_2_, the midguts of all sampled workers were used to extract total RNA with TRIzol immediately after the sampling. Total RNA of 15 infected and 15 control workers were pooled separately daily. Six mRNA libraries were constructed for the infected workers (one mRNA sequence library for each post infection day). Additionally, six mRNA libraries were constructed for the pooled control workers. Since *N*. *ceranae* targets the epithelial cells of the midgut, we could quantify the expression level of transcripts of both host and parasite from infected workers.

### RNA-seq data analysis

Over 60 million reads (100 nucleotides per read) were generated from each mRNA library. The mRNA sequencing reads were aligned to the host and parasite reference genomes by the CLC Genomics Workbench [19]. After trimming adaptors, two mismatches are allowed with deletion cost of three, insertion cost of three, length fraction of 0.8, similarity fraction of 0.8 and maximum 10 hits for a read. For the control workers, reads were mapped to honey bee assembly Amel 4.5 [20]. For the infected workers, reads were aligned to both honey bee assembly Amel4.5 and *N*. *ceranae* assembly ASM18298v1 simultaneously. In order to assess the mis-assignment of the reads between the host and parasite, we re-aligned the reads that previously mapped to the host back to the parasite genome. On average, 10 out of 1 million reads were mapped to both host and parasite. The impact of these shared reads on quantifying gene expression is miniscule, and these reads were excluded from different gene expression statics. For the honey bee host, the impact of infection was analyzed by comparing gene expression levels between uninfected and infected bees for each time interval. The original counts were normalized with weighted trimmed mean of M-values (TMM) to calculate relative expression levels of mRNA to calculate statistically differentially expressed transcripts edgeR Package [21]. As there is no biological replicate, the common dispersion of 0.02 was used. For the parasite, maSigpro package was used to analyze time series gene expression patterns cross the reproduction cycle [22].

### *N*. *ceranae* SNP position identification and annotation

We combined all the reads aligned to *N*. *ceranae* genome from of all six libraries to search expression variations (> 100 coverage). Single nucleotide polymorphism (SNP) positions were identified by PICARD–GATK—SNPEFF pipeline [23]. In order to compare the selective pressure on the parasite genetic diversity, we identified SNP positions with the parasite collected from the same apiary in 2007. Raw reads (454 sequencing) used to assemble the *N*. *ceranae* genome were downloaded from NCBI [7]. The identified SNP were annotated by snpEff package [24]. We were able to measure pairwise differences in SNP counts for 1479 predicted genes in the *N*. *ceranae* genome for which conservative SNP positions had been identified in both 2007 and 2013. Mean SNP counts were estimated, along with means for non-synonymous and synonymous SNP positions. Paired analysis of the same sets of 1479 genes were used to avoid biases due to gene length, i.e., the gene space used for SNP analyses in both current and historic samples was identical. In addition, for those gene models (n = 465) containing five or more SNP positions in both 2007 and 2013, we regressed total SNP count, non-synonymous, and synonymous counts between samples. We also estimated the ratio of non-synonymous to total SNP positions for each of the 1479 gene models to assess selection on proteins [25].

### Verification gene expression levels with quantitative real time PCR

Since antimicrobial peptides (AMPs) have been reported to respond towards *Nosema* infection, albeit in contrasting ways across different studies [13,17], two host AMPs, *Apidaecin* and *Hymenoptaecin*, were selected alongside two parasite genes, *Dicer* and *PIWI* to verify the sequencing data by RT-qPCR. We chose *Nosema Dicer* and *PIWI*, since they are involved in the synthesis of small interfering RNAs, arguably candidates for determining whether *N*. *ceranae* has a functional RNAi pathway. Five infected and uninfected honey bees were pooled as one replicate respectively. Three replicates of each time point were used for quantitative real time PCR assays from 1 dpi to 6 dpi. The relative gene expression level of the host was standardized to GAPDH which is known to remain stable during *Nosema* infection [14]. The relative Ct value of two parasite genes were used for the validation of the gene expression of the two techniques. The amplification efficiency of the primers were calculated by qpcR package [26]. T-tests were used to determine the significant difference of relative Ct values.

## Results

### Time series expression of parasite genes

**Fig 1:**
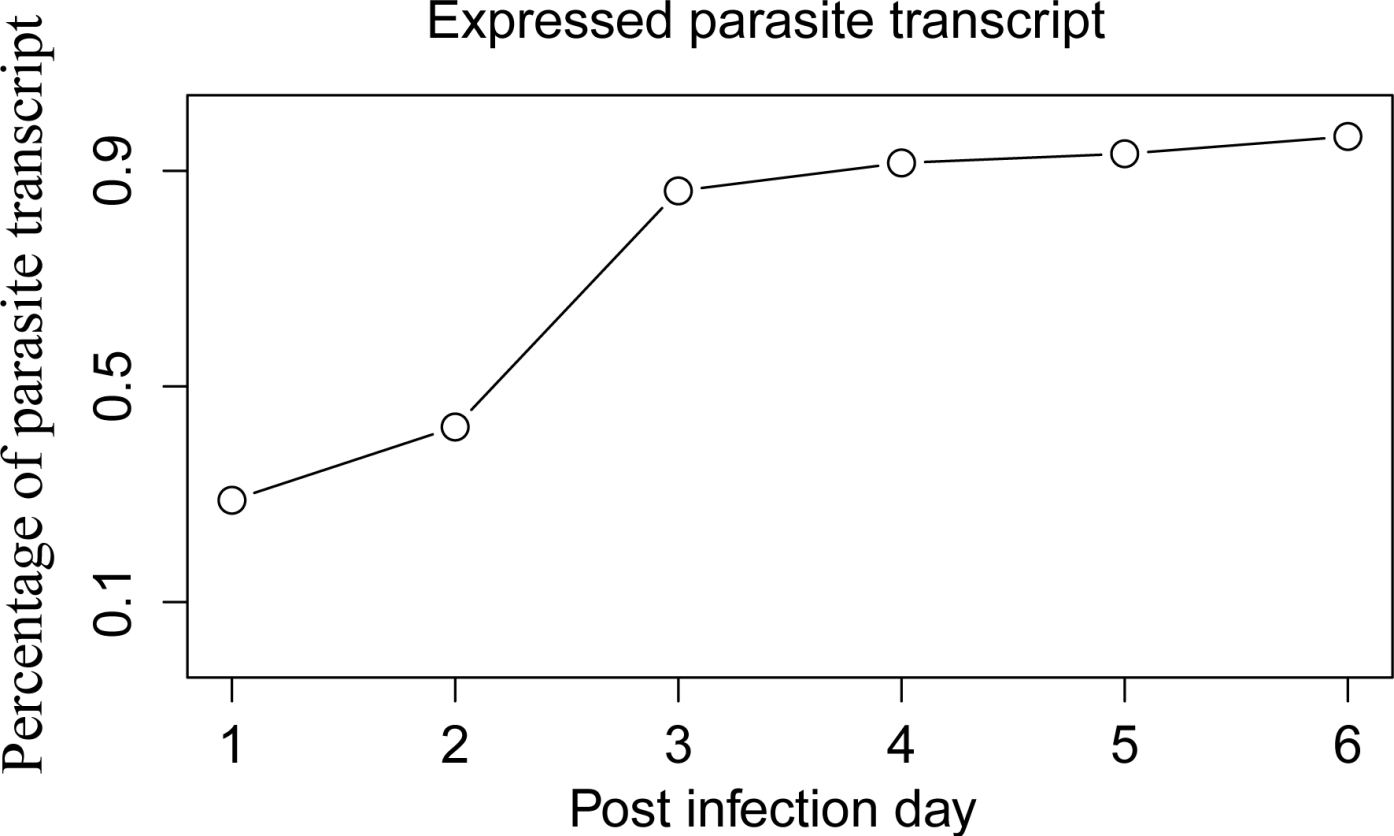
Percentage of total *N*. *ceranae* transcripts detected over the infection period. Over 28% of *N*. *ceranae* transcripts were detected to express as early as 1 dpi. The expressed transcripts increased dramatically from 42.5% at 2 dpi to 91.5% at 3 dpi, followed by a steady increase to 96.4% at 6 dpi. The largest increase in the expressed transcripts was between 2 dpi and 3 dpi, which might reflect an increase in meront reproduction and sporonts formation.

We detected 1988 different genes from the total predicted *N*. *ceranae* gene set (n = 2060), across all libraries during the experimental period. Expressed parasite transcripts increased from 28.9% of the predicted genes at 1 dpi to 96.4% at 6 dpi (Fig 1). Total reads mapped to the *N*. *ceranae* genome increased from 5*10^3^ at day 1 dpi to 5*10^6^ at 6 dpi, confirming fast proliferation at later phases of *N*. *ceranae* infection.

**Fig 2:**
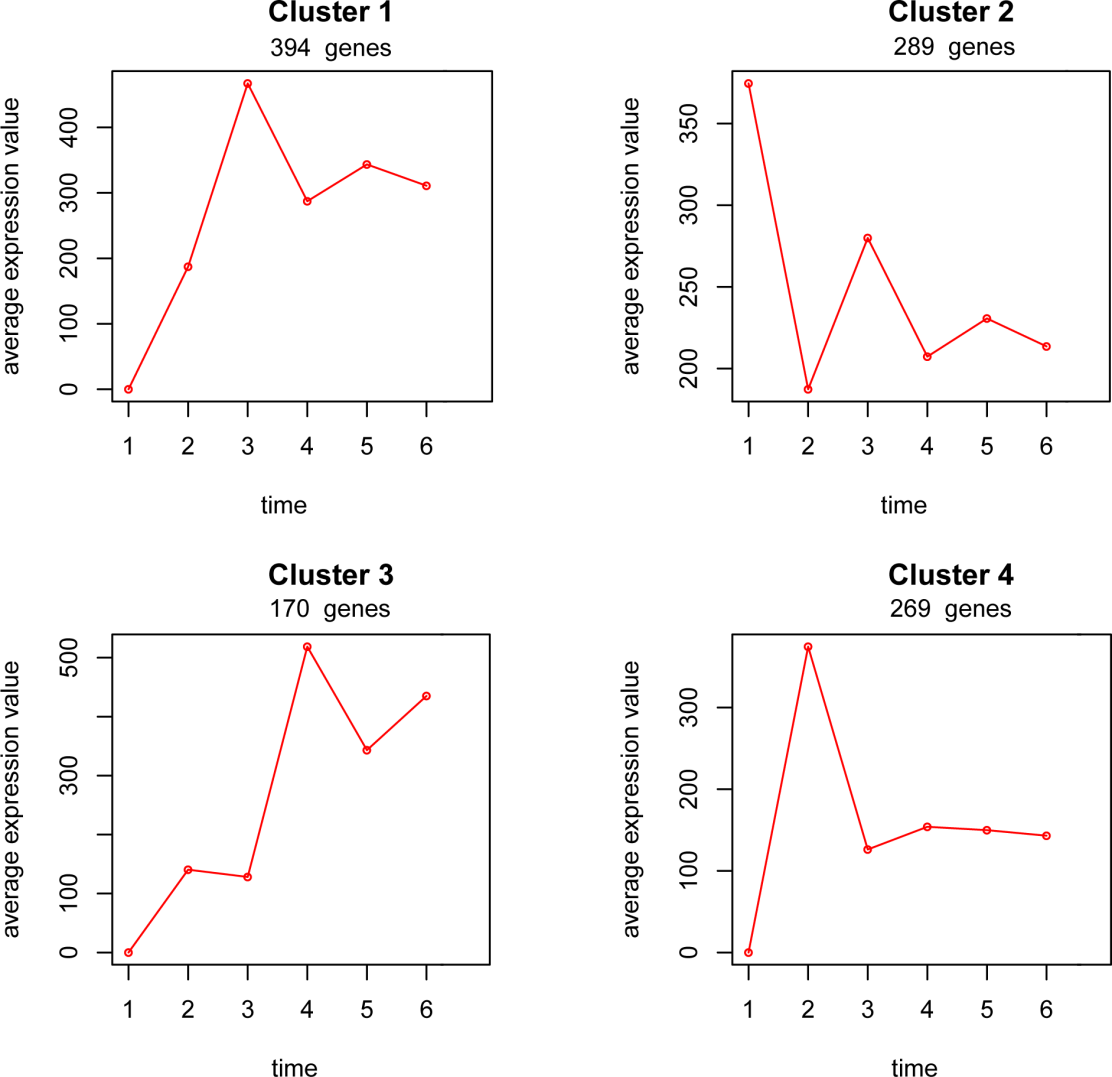
*N*. *ceranae* gene expression pattern during the infection period. There were 1122 genes significantly differentially expressed during the infection period, clustering into 4 expression patterns based on the expression levels as above. The X axis represents post-infection day and the Y axis represents average counts of clustered genes.

In total, 1122 parasite genes were significantly differentially expressed at some point during the infection period (S1 File), clustering into 4 different gene expression patterns based on the normalized gene expression level (Fig 2). There were 595 parasite genes expressed as early as 1 dpi, including RNA polymerase, DNA polymerase, ribosomal proteins, APTase, tRNA synthesis, and transporters. Once the sporoplasm of the parasite enters the host cytoplasm, spore replication is initiated. Actin is potentially important to support the structure and vesicle traffic of the membrane. *N*. *ceranae* actins (NCER_101664, NCER_101382, NCER101064, NCER_100589, p < 0.01) were significantly differently expressed over the infection period, particularly for the early infection phase (cluster 1 and cluster 2). The *Nosema* cell differentiation regulator (NCER_101467, cluster 4, p < 0.001) and histones (NCER_101385, cluster 4, p < 0.001) were highly expressed during the period of highest cell division. Interestingly, thioredoxin (NCER_101950, cluster 3, p < 0.001) and heat shock protein 70 (NCER_101994, cluster 1, p < 0.001) with a predicted signal peptide were also found highly expressed at the cell-division phase.

The expression of core enzymes catalyzing glycolysis was detected as early as 1 dpi. Hexokinase (NCER_101108, cluster 1, p < 0.001) was expressed from 2 dpi onward and this expression increased afterward. Other core enzymes, such as Glucose-6-phosphate isomerase (NCER_100921, p < 0.05), glucose-6-phosphate dehydrogenase (NCER_100052, p > 0.05), fructose-bisphosphate aldolase (NCER_100242, p > 0.05) and triosephosphate isomerase (NCER_100030, p > 0.05) were all detected since 3 dpi. ATP/ADP transporter (NCER_100306, p > 0.05) and ABC transporter (NCER_100674, p > 0.05) were highly expressed throughout the infection period.

### Putative *N*. *ceranae* virulence genes

The apoptosis inhibitor gene (NCER_100918, cluster 4, p < 0.001) is interesting because it could inhibit apoptosis by binding activated caspase. Expression of this apoptosis inhibitor was detected from 2 dpi onward. The highest expression level occurred on 2 dpi and then decreased during the experimental period. In addition, genes for two proteins involved in small regulatory RNA production, *Argonaut* (NCER_101240, cluster 2, p < 0.001) and *Dicer* (NCER_100079, p > 0.05), were expressed as early as 1 dpi. The toxin ricin (NCER_101673) was expressed from 2 dpi onward.

### Single Nucleotide Polymorphisms (SNPs) and loss of SNPs in *N*. *ceranae* genome

**Fig 3:**
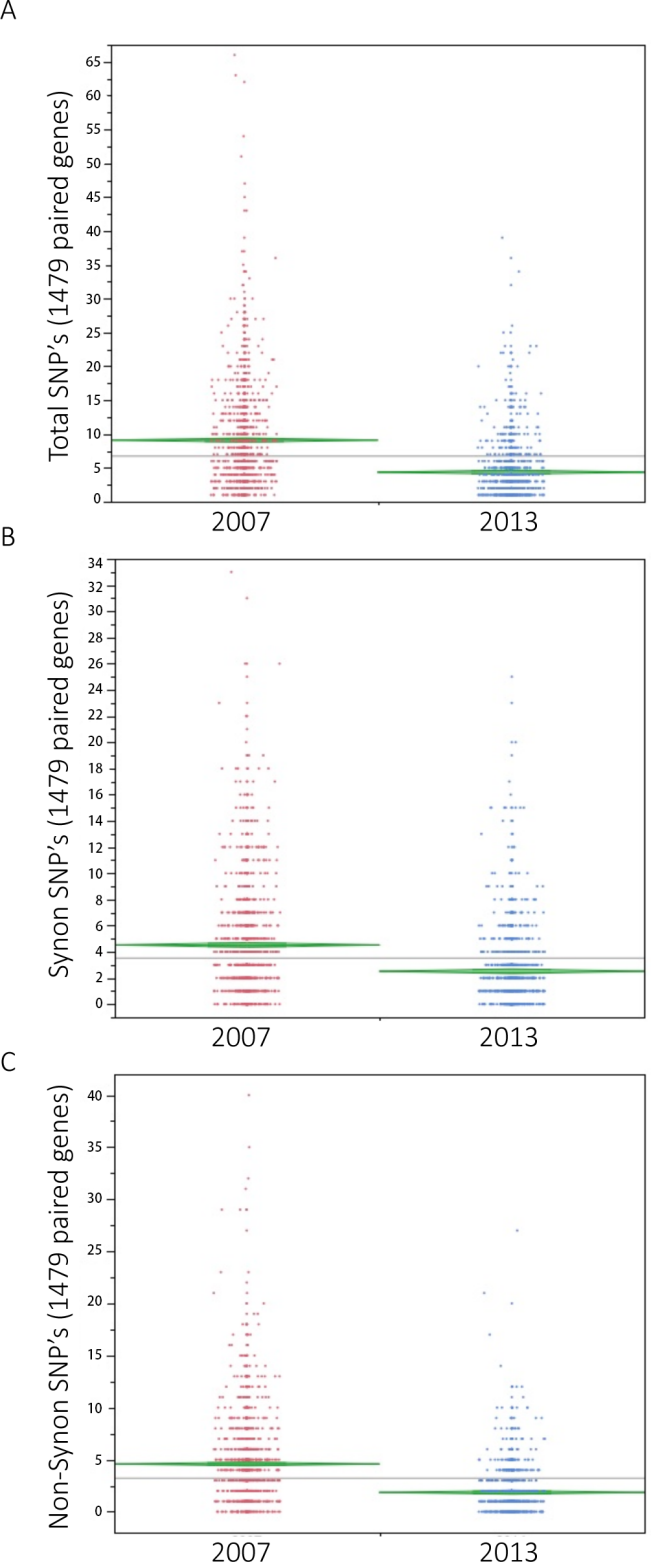
SNP positions within 1479 shared genes in *N*. *ceranae* parasites collected in 2007 and 2013. A: The average number of SNP decreased from 9.1 per gene to 4.36 per gene (p < 0.001). B: Within the total SNP count, the average number of synonymous substitutions decreased from 4.5 in 2007 to 2.81 in 2013. C: The average non-synonomous SNP level dropped from 4.58 in 2007 to 1.85 in 2013. X axis represents two sample groups. Y axis represents number of SNP positions.

Neither introns nor alternative splicing were detected, confirming the *N*. *ceranae* genome is compact and genes generally consist of a single exon. We found substantial variation across the reads at the nucleotide level. 6525 SNP positions were identified from coding DNA sequence (CDS) regions based on our RNA sequencing reads (S2 File). In order to determine the selection direction, we identified the SNP positions from *N*. *ceranae* collected in 2007 from the same apiary. Its DNA sequencing (454 Life Science Roche) data has been used to assemble *N*. *ceranae* genome and downloaded from NCBI (Fig A in S6 File). Over 1 million reads were re-aligned to *N*. *ceranae* genome with 35 times coverage. Out of 6525 SNP positions identified from samples in 2013, 4405 SNP positions were found at the exact physical location from samples in 2007. The chance is low (y = 1/(4405*7.86*10^6^) to call an incorrect SNP at the exact same positions from two sample sets. The number of SNP positions within coding sequence (CDS) regions dramatically dropped from 16555 to 6525 (p < 0.001) and also the number of genes containing SNP dropped from 2018 to 1516 after six years infection (p < 0.001). Out of 1516 genes, 1479 genes had SNP in parasites collected in both 2007 and 2013. Within the same set of 1479 genes, the average number of SNP decreased from 9.1 per gene to 4.36 per gene (Fig 3A) and the density of SNP decreased from 122 base pairs per SNP in 2007 to 254 base pairs per SNP in 2013 (p < 0.001). Within the total SNP count, the number of synonymous SNP substitutions decreased at a slower rate than did non-synonymous SNP substitutions. The average number of synonymous substitutions in 2007 was 4.52±0.01 (Mean±SE), which decreased to 2.81±0.73 (Mean±SE) in 2013 (Fig 3B), while the non-synonymous SNP level decreased from 4.58 ± 0.11to 1.85 ± 0.59 (Fig 3C). Accordingly the ratio of non-synonymous to total SNP dropped from 0.50 ± 0.0068 (Mean±SE) to 0.43 ± 0.0091(Mean±SE) between 2007 and 2013 (p < 0.001). When confining gene pairs to those showing at least 5 distinct SNP positions in each gene (n = 465), a linear regression indicates that paired genes in 2013 showed 55% of the synonymous SNP positions found in 2007 while only 44% of the non-synonymous SNP positions were present in 2013 (p < 0.01, Fig B in S6 File). The quality score of each SNP position of two sample points were available in S4 and S5 Files.

### Expression of host during infection

**Fig 4:**
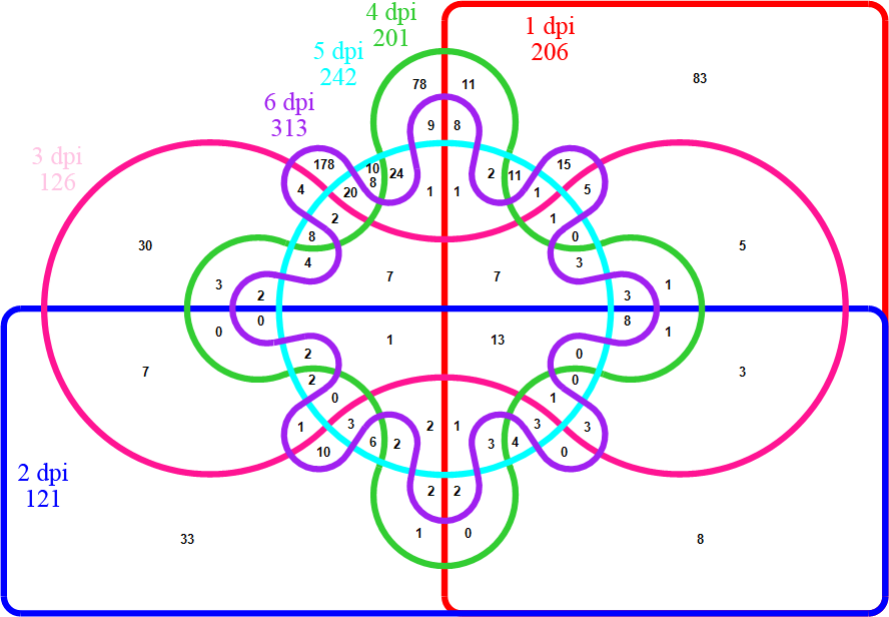
Six-way Venn diagram showing the distribution of shared significantly regulated host transcripts during the infection period, relative to control honey bees of the same age. Numbers of clusters are provided in the interactions. The total number of significantly regulated host transcripts is provided for each day post infection.

After *N*. *ceranae* infection, expression levels of 206, 121, 126, 201, 242, and 313 honey bee transcripts were significantly differently expressed between infected bees and controls at time points from 1 dpi to 6 dpi respectively (S3 File). Out of those, 13 transcripts were significantly regulated in the same direction during the entire infection period (Fig 4).

The antimicrobial peptides (AMPs) *Abaecin* (LOC406144), *Apidaecin* (*Apid73*), *Defensin* (*Def1*) and *Hymenoptaecin* (LOC406142) were all significantly over-expressed in infected bees compared with control bees at 1 dpi (p < 0.01). The expression levels of *Abaecin* (p > 0.05), *Apidaecin* (p < 0.01) and *Defensin* (p < 0.01) were slightly decreased at 2 dpi, but still up-regulated in the infected bees compared with controls. However, the expression level of *Hymenoptaecin* was significantly down-regulated in infected bees at 2 dpi (p < 0.01). AMP expression levels increased again in infected bees at 3 and 4 dpi. Interestingly, *Abaecin* (p > 0.05), and *Defensin* (p < 0.05) were down-regulated at 5 dpi. *Defensin* (p > 0.05), *Abaecin* (p < 0.01), *Apidaecin* (p < 0.01) and *Hymenoptaecin* (p < 0.01) were again significantly up-regulated at 6 dpi (Table 1).

**Table 1:** Immunity related genes and involved pathways.

Pathway	Gene	1 dpi	2 dpi	3 dpi	4 dpi	5 dpi	6 dpi
Toll	Abaecin	up**	up	up *	up *	down	up **
Toll	Apidaecin	up*	up**	up**	up**	up	up**
Toll	Defensin	up**	up**	up	up	down*	up
Toll	Hymenoptaecin	up**	down**	up**	up**	up	up**
Toll	PPO	down*	down	up	down	up	up
JAK/STAT	TEP7	down	down	down	up	up**	up
Apoptosis	reactive oxygen species modulator 1-like	down**	down**	down**	down**	down**	down**

Dpi means day post infection.

Up indicates the gene expression level is up-regulated in the infected honey bees compared with uninfected honey bees.

Down indicates the gene expression level is down-regulated in the infected honey bees compared with uninfected honey bees.

* indicates the regulation is significantly at FDR < 0.05 and

** indicates the regulation is significantly at FDR < 0.01.

Besides the AMPs, the melanization agent prophenoloxidase subunit A3 (*PPO*) was significantly down-regulated in infected bees at early infection (1 dpi, p < 0.01) and then showed a similar expression with the control bees. Thiolester-containing protein 7 (*TEP7*) is an antimicrobial protein of JAK/STAT pathway. The expression level of *TEP7* tended to be down-regulated from 1 to 3 dpi and then up-regulated from 4 to 6 dpi, although statistical significance was only detected at 5 dpi (p < 0.01) (Table 1).

### RNA interference pathway responses of the host

The expression level of transcripts for the honey bee *Dicer* protein (Dcr-1) tended to be higher for infected bees than controls at 1 dpi. The *R2D2* orthologue (*PRM1*) showed significant up-regulation at 1 dpi for infected bees (p < 0.01) after which expression levels were similar between infected and control bees, a pattern shared by *Argonaute 2* (AGO2). The expression level of helicases (*Melt*, *bel*, *DDX17*, LOC726768, LOC100576828) involved in maintaining the structure of the RNA induced silencing complex was consistently high, but did not significantly differ between the infected and control bees. The expression level of *Aubergine* was down-regulated in infected bees when compared to control bees, at two days post-infection, after which expression levels for this protein were similar between infected and control bees.

### Apoptosis pathway responses of the host

Apoptosis can be activated by either blood cells or mitochondria. As there is no report suggesting the existence of hemocytes in the honey bee midgut, mitochondria oriented apoptosis is presumably the mechanism to respond to *N*. *ceranae* infection, if there is a response. The key genes involved in apoptosis were expressed from 1 dpi onward. Anti-apoptotic (LOC409189, LOC100579020) as well as pro-apoptotic (LOC411613) genes in BCL-2 family showed similar expression levels between infected and control honey bees. Caspases (LOC411381, LOC724390, LOC412235, LOC10057648), inhibitor of apoptosis (LOC726172, LOC726899, *lap2*, *Det*), tumor suppress protein P53-like (LOC10057712, LOC10057853) and cytochrome c (*CytC*) all had no significant differences between infected and control bees. However the expression level of reactive mitochondrial oxygen species modulator (LOC727012) was significantly down regulated for all six days post infection (p < 0.01) (Table 1).

### Quantitative real-time PCR verification

**Fig 5:**
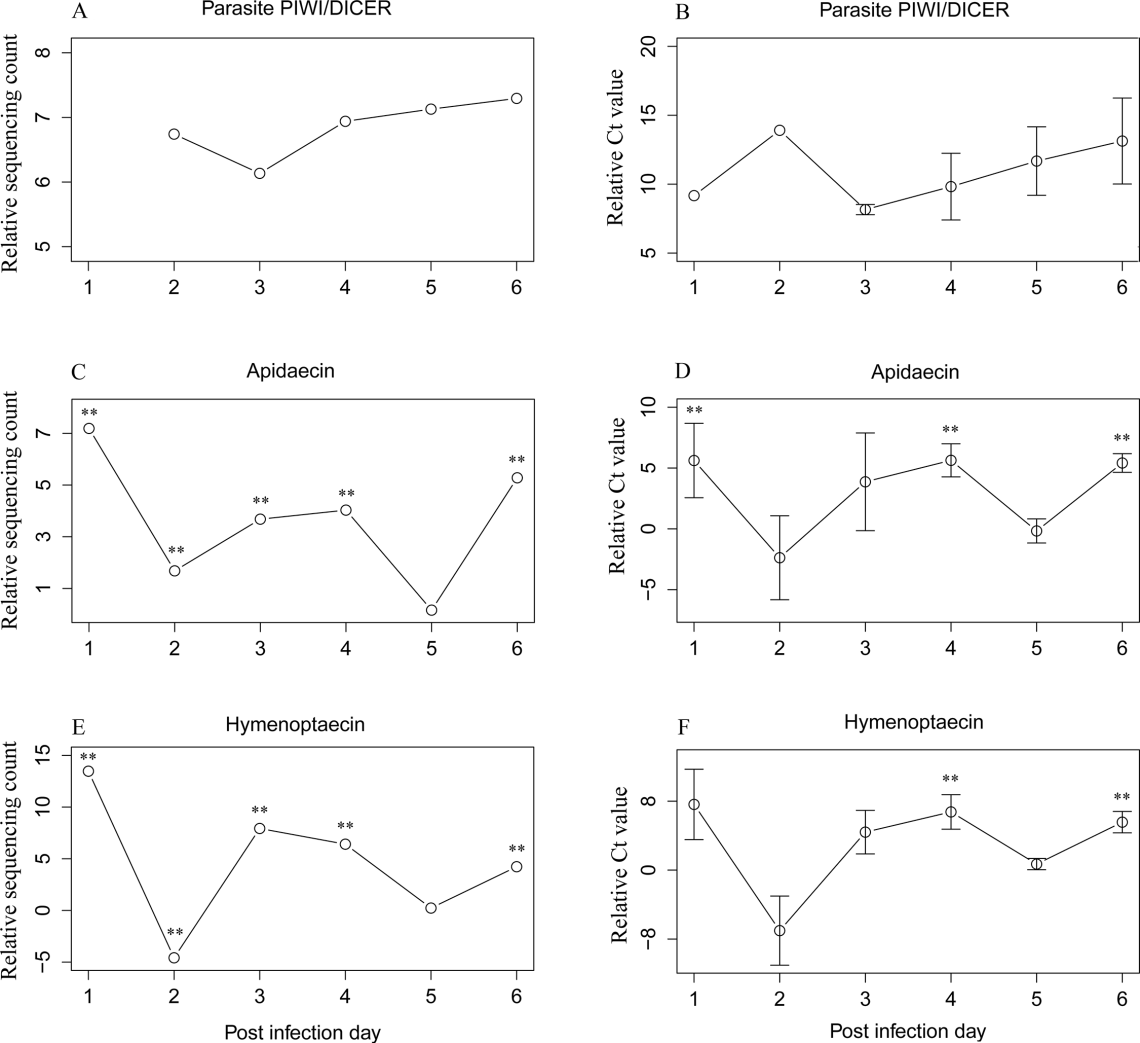
**(A) Count ratios derived from mRNA sequencing for parasitic PIWI and Dicer**. Y = Log((C^PIWI^/C^DICER^), 2), where C^PIWI^ represents normalized counts of parasitic gene PIWI and C^DICER^ represents normalized counts of parasitic gene DICER. The expression of Dicer was only detected starting at 2 dpi, hence data for 1 dpi is missing. (B) Real time quantitative PCR originated expression value of the ratio between parasitic PIWI and Dicer. Y = Log((Ct^DICER^/Ct^PIWI^), 2), where Ct^DICER^ represents the Ct value of gene DICER and Ct^PIWI^ represents the Ct value of gene PIWI. The expression level of PIWI is consistently higher than Dicer with the same expression pattern as sequencing data, which confirmed the validity of the sequencing data. (C) mRNA sequencing originated counts of immune gene *Apidaecin*, which shows significant change over the infection time with a similar expression pattern of qPCR results showed in (D). (E) The mRNA sequencing data of *Hymenoptaecin* again showed significant change over the infection time and shared the same expression pattern as the qPCR results in (F). The consistent expression pattern between the sequencing data and the qPCR results validated the accuracy of the sequencing data. Error bar represents standard error. * represents the significant level at 0.05. ** represents the significant level at 0.01.

The expression levels of selected host and parasite genes were significantly correlated between the two techniques over 6 days post infection (p < 0.05) (Tables A and B in S6 File). *Apidaecin* and *Hymenoptaecin* both showed an up regulation at 1 dpi in the infected bees compared with control bees. The expression levels decreased at 2 dpi, followed by up regulation at 3 dpi and 4 dpi (p < 0.01). The expression level was down regulated again at 5 dpi and up regulated at 6 dpi (p < 0.01). These patterns based on quantitative real time PCR matched RNA sequencing analyses well (Fig 5). The parasite genes *PIWI* and *Dicer* were not detected from control bees yet the expression of PIWI was detected as early as 1 dpi in infected bees. The relative expression level between *PIWI* and *Dicer* is consistent between RNA-seq and RT-qPCR results (Fig 5).

## Discussion

We provide the first time-series gene expression analysis of *N*. *ceranae* parasite during infection, showing over 95% of the gene models predicted for this species are active at some point during infection. Among the 72 undetected *N*. *ceranae* gene models, only 5 have predicted functions based on protein domains, while the remaining 67 are hypothetical with unknown function. These undetected transcripts models might be expressed soon after infection, i.e., prior to our first sampling time point. Alternatively, these might reflect inaccurate gene models, or pseudo-genes that no longer carry a function in this parasite.

Bee responses in relation to *N*. *ceranae* infection were strong during the initial (1 dpi) and late infection phase (6 dpi). In a previous study, midgut expression of 336 honey bee genes was significantly altered by *N*. *ceranae* infection at 7 dpi with genes involved in oxidation reduction being significantly over expressed in the infected bees [27]. Genes involved in transportation and metabolism shown by microarray analyses of fat body and midgut tissues were significantly altered in response to *Nosema* infection [28]. Our data also showed that genes involved in oxidation-reduction processes as well as transporters were significantly up-regulated by infection, which might promote host survival and fuel the parasite reproduction respectively. Moreover, we found that venom proteins were significantly up-regulated at 3 dpi and 4 dpi, followed by a down regulation at 5 dpi and then up regulation again at 6 dpi. Surprisingly, the antimicrobial peptides showed a similar expression pattern. It is unclear why both AMPs and venom proteins showed a general suppression at 5 dpi. As one possible explanation, the reproduction cycle of *N*. *ceranae* is approximately four days. The suppressed expression level therefore might result from the parasite-mediated death of infected honey bee cells. As *Nosema* daughter spores re-enter the midgut lumen, AMPs might be activated again, explaining why AMPs were significantly up-regulated at 1 dpi and 6 dpi. As the RNA was extracted from midgut tissues, part of the expression of genes related with immunity could have been missed after discarding the fat body.

As an intracellular parasite, the nutrients used for *Nosema* replication and growth fueled by ATP come mainly from the host. Glycolysis and oxidative phosphorylation are the main catalytic reactions involved in energy generation. Initially, we expected an up-regulation of honey bee genes involved in glycolysis and oxidative phosphorylation as a way to counter the energetic burden of infection. Surprisingly, we did not find significant regulation of transcripts in either glycolysis or oxidative phosphorylation pathways, despite the constant consumption of ATP by the parasite. The genes encoding core enzymes catalyzing glycolysis were constantly highly expressed for the entire infection period including hexokinase (LOC551005), phosphofructokinase (LOC724724) and pyruvate kinase (LOC552007). The genes encoding the core enzymes that catalyze oxidative phosphorylation were highly expressed as well, specifically citrate synthase (LOC410059), isocitrate dehydrogenase (LOC551276) and succinate dehydrogenase (LOC725566). In the *Encephalitozoon* microsporidia parasite, the total ATP concentration within infected cells was surprisingly not significantly different from uninfected cells in spite of the energy demands imposed by the parasite [29]. ATP concentration regulates the catalytic activity of phosphofructokinase and pyruvate kinase. High ATP concentrations could inhibit glycolysis and oxidative phosphorylation and vice versa. However infected bees do consume more sugar water than control bees to maintain a constant ATP concentration, which indicates the infected bees are indeed energetically parasitized [12]. The variation of catalytic activity of the core enzymes of glycolysis oxidative phosphorylation may not be manifested from the expression level but rather by ATP concentration. Furthermore, the expression levels for all ATP/ADP transporters, ABC transporters and glycolysis enzymes decreased at later infection phases (6 dpi), suggesting that less energy is required during the spore-forming phase

In order to maximize reproduction, *N*. *ceranae* must complete at least one reproductive cycle before killing the infected host. Genes involved in reducing oxidative damage, protein folding and promoting cell growth were detected early during infection. Indeed, in spite of abundant daughter spores filling the cytoplasm, infection does not induce either apoptosis or necrosis before completing the reproductive cycle [10,30,31]. *N*. *ceranae* most likely regulates host cell apoptosis at two levels: by blocking the activated caspase enzymes and by blocking the releasing of cytochrome c from the mitochondria. Caspases play central roles in apoptosis. Even though caspases are activated, apoptosis still could be regulated by inhibitors of apoptosis (IAPs), which could bind and inactivate the caspases to suppress the apoptosis [32]. The *N*. *ceranae* genome contains one IAPs gene (NCER_100918), which was expressed in infected bees as early as 2 dpi. The Bcl2 proteins, a family containing both anti-apoptotic and pro-apoptotic proteins, regulate the release of cytochrome c and other inter-membrane proteins. In principle, the anti-apoptotic proteins of *Nosema* could bind to host pro-apoptotic proteins to balance levels of apoptosis, i.e., once host genes coding pro-apoptotic proteins are over-expressed, apoptosis is triggered [33]. From our data, we did not find significant differences among Bcl2 proteins. However, the reactive oxygen species modulator 1-like gene (ROS) was significantly down-regulated in infected bees compared with non-infected bees for all six dpi. Over expression of ROS is known to activate the pro-apoptotic Bcl-2, thereby inducing apoptosis. On the other hand, reduced ROS could promote cell proliferation and protect the integrity of mitochondrial membranes, limiting apoptosis [34]. Maintaining functionality of host mitochondria is vital for parasite energetics and reproductive success. Therefore, protecting host mitochondrial membrane integrity to prevent the release of cytochrome c is a likely method used by the parasite to regulate host apoptosis.

Ricin is a cytotoxic protein with a lectin domain, which could inhibit protein synthesis by interfering with ribosomes [35,36]. As we noticed, a Ricin-type beta-trefoil lectin protein-coding gene was expressed from the parasite transcript as early as 2 dpi. Moreover, this ricin protein has a predicted signal peptide, which suggests it might be secreted into the host’s cytoplasm to inhibit host protein synthesis to slow down the infected cell metabolism. In a fish-infecting microsporidian parasite, *Spraguea lophii*, a secreted ricin protein has been identified as a virulence protein [37]. From our data, the expression levels of the ribosomal proteins of the host were generally significantly suppressed in the infected bees compared with the control bees. The parasite may secrete the toxic ricin into the bee’s cytoplasm to reduce the host’s protein synthesis, which could be a general parasitism method used by microsporidia [1,7,37,38].

*N*. *ceranae* has become a wide spread parasite in the honey bee *A*. *mellifera* since the first report in 2006 [39,40]. After a worldwide expansion into this new host, *N*. *ceranae* still shows a high level of genetic diversity, which was even higher within colonies than among colonies [15,41]. However, it is unclear how selection pressures during this dispersal have affected *N*. *ceranae*. By comparing the *N*. *ceranae* spores collected in 2013 with those collected in 2007, we confirm that *N*. *ceranae* in our research apiary maintains substantial genetic variation. Still, we found that both total number of SNP positions cross all homologous regions and the number of SNP positions per CDS were significantly reduced. A disproportionate fraction of this reduction occurred for non-synonymous SNP positions, suggesting purifying selection on *N*. *ceranae* protein-coding genes, which was also suggested from gene analysis [15,42]. A similar phenomenon has also been observed for Deformed wing virus (DWV) in honey bees. The diversity of this virus was significantly reduced after establishing infection and in particular after population growth driven by a biological vector, the parasitic mite *Varroa destructor* [43]. In this case, selection is not based on *de novo* adaption of protein sequences but rather the tremendous success of particular existing strains that were provided a competitive advantage with the arrival of mite vectors. As genetic diversity for *N*. *ceranae* is unexpectedly high within colonies as opposed to among colonies, this reduced genetic diversity might reflect a bottleneck effect due to honey bee colony losses, whereby *Nosema* strains representing a small fraction of that existing variation do especially well under current selective pressures. The gene flow of *N*. *ceranae* is not stopping, which could still substantially changed the current *N*. *ceranae* diversity, as *N*. *ceranae* diversity from Asian honeybees (*Apis cerana* and *Apis florea*) still showed significant differentiation from those obtained from *Apis mellifera* [44]. In conclusion, surviving honey bees and their parasites might be under intense selection for tolerance, since surviving colonies are predominantly the ones spreading *N*. *ceranae*. Alternatively, a few *N*. *ceranae* strains might be dominating infection due to direct selection on this parasite as it co-evolves with its new host species. These hypotheses can be further tested with the SNPs described here and by Pellin et al (2015) [41] and with an explicit understanding of the effects of sequence changes on amino acid sequences for proteins involved in host interactions.

## Supporting Information

S1 File *N*. *ceranae* transcriptome expression data. (XLSX)

S2 File *N*. *ceranae* SNP data. (XLSX)

S3 File *A*. *mellifera* transcriptome data. (XLSX)

S4 File Quality of SNP positions from the sample collected in 2007. (TXT)

S5 File Quality of SNP positions from the sample collected in 2013. (TXT)

S6 File Total SNP distribution along the longest parasite contig (Fig A). Regression between Synonymous and Non-synonymous SNP positions between parasites collected in 2007 and 2013 (Fig B). Ct value of Apidaecin, Hymenoptaecin, Dicer and Piwi (Table A). Normalized counts of Apidaecin, Hymenoptaecin, Dicer and Piwi (Table B).
(DOCX)

## References

[1] Cuomo CA, Desjardins CA, Bakowski MA, Goldberg J, Ma AT, Becnel JJ, et al Microsporidian genome analysis reveals evolutionary strategies for obligate intracellular growth. Genome Res. 2012; 22: 2478–2488. 10.1101/gr.142802.112 22813931 PMC3514677

[2] Bohne W, Bottcher K, Gross U. The parasitophorous vacuole of *Encephalitozoon cuniculi*: Biogenesis and characteristics of the host cell-pathogen interface. Int J Med Microbiol. 2011; 301: 395–399. 10.1016/j.ijmm.2011.04.006 21550847

[3] Fries I. *Nosema ceranae* in European honey bees (*Apis mellifera*). J Invertebr Pathol. 2010; 103: S73–S79. 10.1016/j.jip.2009.06.017 19909977

[4] Fokin SI, Di Giuseppe G, Erra F, Dini F. Euplotespora binucleata n. gen., n. sp (Protozoa: Microsporidia), a parasite infecting the hypotrichous ciliate Euplotes woodruffi, with observations on microsporidian infections in Ciliophora. J Eukaryot Microbiol. 2008; 55: 214–228. 10.1111/j.1550-7408.2008.00322.x 18460159

[5] Gisder S, Mockel N, Linde A, Genersch E. A cell culture model for *Nosema ceranae* and *Nosema* apis allows new insights into the life cycle of these important honey bee-pathogenic microsporidia. Environ Microbiol. 2011; 13: 404–413. 10.1111/j.1462-2920.2010.02346.x 20880328

[6] Chen YP, Pettis JS, Zhao Y, Liu X, Tallon LJ, Sadzewicz LD, et al Genome sequencing and comparative genomics of honey bee microsporidia, *Nosema apis* reveal novel insights into host-parasite interactions. BMC Genomics. 2013; 14. 10.1186/1471-2164-14-451 PMC3726280 23829473

[7] Cornman RS, Chen YP, Schatz MC, Street C, Zhao Y, Desany B, et al Genomic analyses of the microsporidian *Nosema ceranae*, an emergent pathogen of honey bees. PLoS Path. 2009; 5. 10.1371/journal.ppat.1000466 PMC2685015 19503607

[8] Peyretaillade E, El Alaoui H, Diogon M, Polonais V, Parisot N, Biron DG, et al Extreme reduction and compaction of microsporidian genomes. Res Microbiol. 2011; 162: 598–606. 10.1016/j.resmic.2011.03.004 21426934

[9] Burri L, Williams BAP, Bursac D, Lithgow T, Keeling PJ. Microsporidian mitosomes retain elements of the general mitochondrial targeting system. P Natl Acad Sci USA. 2006; 103: 15916–15920. 10.1073/pnas.0604109103 PMC1635103 17043242

[10] Higes M, Juarranz A, Dias-Almeida J, Lucena S, Botias C, Meana A, et al Apoptosis in the pathogenesis of *Nosema ceranae* (Microsporidia: Nosematidae) in honey bees (*Apis mellifera*). Env Microbiol Rep. 2013; 5: 530–536. 23864567 10.1111/1758-2229.12059

[11] Kurze C, Le Conte Y, Dussaubat C, Erler S, Kryger P, Lewkowski O, et al Nosema Tolerant Honeybees (*Apis mellifera*) Escape Parasitic Manipulation of Apoptosis. PloS One. 2015; 10. 10.1371/journal.pone.0140174 PMC4596554 26445372

[12] Mayack C, Naug D. Energetic stress in the honeybee *Apis mellifera* from *Nosema ceranae* infection. J Invertebr Pathol. 2009; 100: 185–188. 10.1016/j.jip.2008.12.001 19135448

[13] Antúnez K, Martín-Hernández R, Prieto L, Meana A, Zunino P, Higes M. Immune suppression in the honey bee (*Apis mellifera*) following infection by *Nosema ceranae* (Microsporidia). Environ Microbiol. 2009; 11: 2284–2290. 10.1111/j.1462-2920.2009.01953.x 19737304

[14] Huang Q, Kryger P, Le Conte Y, Moritz RFA. Survival and immune response of drones of a Nosemosis tolerant honey bee strain towards *N*. *ceranae* infections. J Invertebr Pathol. 2012; 109: 297–302. 10.1016/j.jip.2012.01.004 22285444

[15] Gomez-Moracho T, Maside X, Martin-Hernandez R, Higes M, Bartolome C. High levels of genetic diversity in *Nosema ceranae* within *Apis mellifera* colonies. Parasitology. 2014; 141: 475–481. 10.1017/S0031182013001790 24238365

[16] Dussaubat C, Maisonnasse A, Crauser D, Beslay D, Costagliola G, Soubeyrand S, et al Flight behavior and pheromone changes associated to *Nosema ceranae* infection of honey bee workers (*Apis mellifera*) in field conditions. J Invertebr Pathol. 2013; 113: 42–51. 10.1016/j.jip.2013.01.002 23352958

[17] Schwarz RS, Evans JD. Single and mixed-species trypanosome and microsporidia infections elicit distinct, ephemeral cellular and humoral immune responses in honey bees. Dev Comp Immunol. 2013; 40: 300–310. 10.1016/j.dci.2013.03.010 23529010

[18] Huang SK, Csaki T, Doublet V, Dussaubat C, Evans JD, Gajda AM, et al Evaluation of cage designs and feeding regimes for honey bee (Hymenoptera: Apidae) laboratory experiments. J Econ Entomol. 2014; 107: 54–62. 24665684 10.1603/ec13213

[19] Frey KG, Herrera-Galeano JE, Redden CL, Luu TV, Servetas SL, Mateczun AJ, et al Comparison of three next-generation sequencing platforms for metagenomic sequencing and identification of pathogens in blood. BMC Genomics. 2014; 15: 96 10.1186/1471-2164-15-96 24495417 PMC3922542

[20] Elsik CG, Worley KC, Bennett AK, Beye M, Camara F, Childers CP, et al Finding the missing honey bee genes: lessons learned from a genome upgrade. BMC Genomics. 2014; 15. 10.1186/1471-2164-15-86 PMC4028053 24479613

[21] Robinson MD, McCarthy DJ, Smyth GK. edgeR: a Bioconductor package for differential expression analysis of digital gene expression data. Bioinformatics. 2010; 26: 139–140. 10.1093/bioinformatics/btp616 19910308 PMC2796818

[22] A. C, M.J. N. maSigPro: Significant Gene Expression Profile Differences in Time Course Microarray Data. R package version. 1.40.0; http://bioinfo.cipf.es/.

[23] Van der Auwera GA, Carneiro MO, Hartl C, Poplin R, Del Angel G, Levy-Moonshine A, et al From FastQ data to high confidence variant calls: the Genome Analysis Toolkit best practices pipeline. Curr Protoc Bioinformatics. 2013; 11: 11 10 11–11 10 33. 10.1002/0471250953.bi1110s43 PMC4243306 25431634

[24] Cingolani P, Platts A, Wang LL, Coon M, Nguyen T, Wang L, et al A program for annotating and predicting the effects of single nucleotide polymorphisms, SnpEff: SNPs in the genome of *Drosophila melanogaster* strain w(1118); iso-2; iso-3. Fly. 2012; 6: 80–92. 10.4161/fly.19695 22728672 PMC3679285

[25] Mcdonald JH, Kreitman M. Adaptive Protein Evolution at the Adh Locus in Drosophila. Nature. 1991; 351: 652–654. 1904993 10.1038/351652a0

[26] Ritz C, Spiess AN. qpcR: an R package for sigmoidal model selection in quantitative real-time polymerase chain reaction analysis. Bioinformatics. 2008; 24: 1549–1551. 10.1093/bioinformatics/btn227 18482995

[27] Dussaubat C, Brunet JL, Higes M, Colbourne JK, Lopez J, Choi JH, et al Gut Pathology and Responses to the Microsporidium *Nosema ceranae* in the Honey Bee *Apis mellifera*. PloS One. 2012; 7. 10.1371/journal.pone.0037017 PMC3356400 22623972

[28] Holt HL, Aronstein KA, Grozinger CM. Chronic parasitization by *Nosema* microsporidia causes global expression changes in core nutritional, metabolic and behavioral pathways in honey bee workers (*Apis mellifera*). BMC Genomics. 2013; 14. 10.1186/1471-2164-14-799 PMC4046765 24245482

[29] Scanlon M, Leitch GJ, Visvesvara GS, Shaw AP. Relationship between the host cell mitochondria and the parasitophorous vacuole in cells infected with *Encephalitozoon* microsporidia. J Eukaryot Microbiol. 2004; 51: 81–87. 15068269 10.1111/j.1550-7408.2004.tb00166.x

[30] del Aguila C, Izquierdo F, Granja AG, Hurtado C, Fenoy S, Fresno M, et al *Encephalitozoon* microsporidia modulates p53-mediated apoptosis in infected cells. Int J Parasitol. 2006; 36: 869–876. 16753166 10.1016/j.ijpara.2006.04.002

[31] Scanlon M, Leitch GJ, Shaw AP, Moura H, Visvesvara GS. Susceptibility to apoptosis is reduced in the microsporidia-infected host cell. J Eukaryot Microbiol. 1999; 46: 34S–35S. 10519237

[32] LaCasse EC, Baird S, Korneluk RG, MacKenzie AE. The inhibitors of apoptosis (IAPs) and their emerging role in cancer. Oncogene. 1998; 17: 3247–3259. 9916987 10.1038/sj.onc.1202569

[33] Luo X, Budihardjo I, Zou H, Slaughter C, Wang XD. Bid, a Bcl2 interacting protein, mediates cytochrome c release from mitochondria in response to activation of cell surface death receptors. Cell. 1998; 94: 481–490. 9727491 10.1016/s0092-8674(00)81589-5

[34] Simon HU, Haj-Yehia A Fau—Levi-Schaffer F, Levi-Schaffer F. Role of reactive oxygen species (ROS) in apoptosis induction. Apoptosis. 2000; 5: 415–418. 11256882 10.1023/a:1009616228304

[35] Wu JH, Singh T, Herp A, Wu AM. Carbohydrate recognition factors of the lectin domains present in the Ricinus communis toxic protein (ricin). Biochimie. 2006; 88: 201–217. 16140451 10.1016/j.biochi.2005.07.007

[36] Endo Y, Mitsui K, Motizuki M, Tsurugi K. The mechanism of action of ricin and related toxic lectins on eukaryotic ribosomes—the site and the characteristics of the modification in 28-S robosomal RNA casued by the toxins. J Biol Chem. 1987; 262: 5908–5912. 3571242

[37] Campbell SE, Williams TA, Yousuf A, Soanes DM, Paszkiewicz KH, Williams BAP. The Genome of *Spraguea lophii* and the Basis of Host-Microsporidian Interactions. PLoS Genet. 2013; 9. 10.1371/journal.pgen.1003676 PMC3749934 23990793

[38] Pan GQ, Xu JS, Li T, Xia QY, Liu SL, Zhang GJ, et al Comparative genomics of parasitic silkworm microsporidia reveal an association between genome expansion and host adaptation. BMC Genomics. 2013; 14. 10.1186/1471-2164-14-186 PMC3614468 23496955

[39] Fries I, Martin R, Meana A, Garcia-Palencia P, Higes M. Natural infections of *Nosema ceranae* in European honey bees. J Apic Res. 2006; 45: 230–233.

[40] Higes M, Martin R, Meana A. *Nosema ceranae*, a new microsporidian parasite in honeybees in Europe. J Invertebr Pathol. 2006; 92: 93–95. 16574143 10.1016/j.jip.2006.02.005

[41] Pelin A, Selman M, Aris-Brosou S, Farinelli L, Corradi N. Genome analyses suggest the presence of polyploidy and recent human-driven expansions in eight global populations of the honeybee pathogen Nosema ceranae. Environ Microbiol. 2015. 10.1111/1462-2920.12883 25914091

[42] Li Z, Hao YJ, Wang LL, Xiang H, Zhou ZY. Genome-Wide Identification and Comprehensive Analyses of the Kinomes in Four Pathogenic Microsporidia Species. PloS One. 2014; 9. 10.1371/journal.pone.0115890 PMC4280135 25549259

[43] Martin SJ, Highfield AC, Brettell L, Villalobos EM, Budge GE, Powell M, et al Global Honey Bee Viral Landscape Altered by a Parasitic Mite. Science. 2012; 336: 1304–1306. 10.1126/science.1220941 22679096

[44] Gomez-Moracho T, Bartolome C, Bello X, Martin-Hernandez R, Higes M, Maside X. Recent worldwide expansion of *Nosema ceranae* (Microsporidia) in *Apis mellifera* populations inferred from multilocus patterns of genetic variation. Infect Genet Evol. 2015; 31: 87–94. 10.1016/j.meegid.2015.01.002 25583446

