# Supplementary material for: Host-Parasite Interactions and Purifying Selection in a Microsporidian Parasite of Honey Bees
Source: PLoS One. 2016 Feb 3;11(2):e0147549. doi: 10.1371/journal.pone.0147549 (PMC4739525; doi:10.1371/journal.pone.0147549)
Supplement: S6 File — Total SNP distribution along the longest parasite contig (Fig A). Regression between Synonymous and Non-synonymous SNP positions between parasites collected in 2007 and 2013 (Fig B). Ct value of Apidaecin, Hymenoptaecin, Dicer and Piwi (Table A). Normalized counts of Apidaecin, Hymenoptaecin, Dicer and Piwi (Table B). (DOCX) [file pone.0147549.s006.docx]

**Supplementary material**

Figure A SNP regions in the largest contig of *N. ceranae* genome. Black dots represent the SNP distribution within CDS in 2007. Red circles represent SNP distribution within CDS in 2013.

A


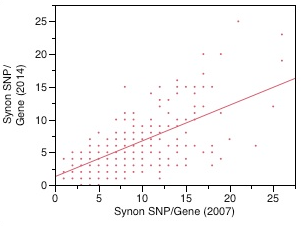


Synon SNP/Gene (2013)

Synon SNP/Gene (2007)

B


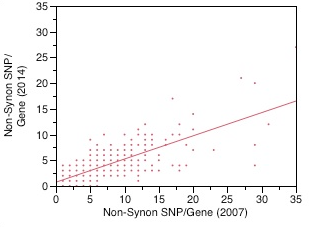


Non-Synon SNP/Gene (2013)

Non-Synon SNP/Gene (2007)

Figure B Regression between Synonymous and Non-synonymous SNP substitution between *N. ceranae* parasite collected in 2007 and 2013. Linear regression indicates that paired genes in 2014 showed 55% of the synonomous SNP’s found in 2007 (SE on slope = 0.026, A) while only 44% of the non-synonomous SNP’s (SE on slope = 0.022, B)  were present in 2013 (difference in slopes significant at p < 0.01)

Table A Ct value of Apidaecin, Hymenptacin, Dicer and Piwi. Eff represents PCR amplification efficiency. Mean±Standard Error

| RT-qPCR |  | GDPH | Apidaecin | Hymenptacin | Dicer | PIWI |
| --- | --- | --- | --- | --- | --- | --- |
| Infection | eff | 1.8976 | 1.8503 | 1.7744 | 1.8855 | 1.8282 |
|  | Day1 | 25.01±0.59 | 33.03±2.9 | 22.21±4.29 | 40.82 | 34.01±0.95 |
|  | Day2 | 27.56±3.84 | 31.64±1.3 | 25.62±1.98 | 42.56 | 32.81±2.28 |
|  | Day3 | 26.28±3.6 | 31.30±1.84 | 25.11±3.09 | 32.06±1.94 | 28.47±4.24 |
|  | Day4 | 26.33±2.6 | 34.21±1.48 | 28.19±0.87 | 35.22±3.9 | 25.10±1.01 |
|  | Day5 | 23.4±0.6 | 34.9±1.42 | 28.4±0.98 | 33.39±2.02 | 21.68±1.01 |
|  | Day6 | 23.92±0.92 | 31.23±0.93 | 26.21±0.52 | 33.6±3.67 | 20.24±0.37 |
| Control | Day1 | 26.1±1.81 | 38.8±1.62 | 32.66±1.85 |  |  |
|  | Day2 | 28.11 | 29.54 | 17.74 |  |  |
|  | Day3 | 23.7±0.35 | 32.97±0.6 | 27.56±0.56 |  |  |
|  | Day4 | 22.89±0.31 | 36.97±0.5 | 32.51±0.88 |  |  |
|  | Day5 | 22.44±0.4 | 33.67±0.65 | 28.18±0.86 |  |  |
|  | Day6 | 22.22±0.4 | 35.56±0.63 | 31.03±0.82 |  |  |

Table B Normalized counts of Apidaecin, Hymenptacin, Dicer and Piwi.

| RNAseq |  | Apidaecin | Hymenptacin | Dicer | PIWI |
| --- | --- | --- | --- | --- | --- |
| Infection | Day1 | 115.88 | 3454 | 0 | 12548 |
|  | Day2 | 98.65 | 19 | 93.6 | 10020 |
|  | Day3 | 75.47 | 376.1 | 232.6 | 16339 |
|  | Day4 | 11 | 2.1 | 101.6 | 12474 |
|  | Day5 | 5.93 | 1.2 | 113.1 | 15839 |
|  | Day6 | 45.69 | 3.6 | 94.2 | 17680 |
| Control | Day1 | 0.79 | 0.3 |  |  |
|  | Day2 | 30.89 | 452.8 |  |  |
|  | Day3 | 5.88 | 1.5 |  |  |
|  | Day4 | 0.67 | 0.02 |  |  |
|  | Day5 | 5.31 | 0.98 |  |  |
|  | Day6 | 1.17 | 0.19 |  |  |
|  |  |  |  |  |  |
